# A minimally invasive endoscope assisted retrosigmoid approach for removal of arachnoid cysts in the internal auditory canal: a step by step description

**DOI:** 10.1016/j.bjorl.2019.06.016

**Published:** 2019-08-06

**Authors:** Arianna Di Stadio, Antonio della Volpe, Massimo Ralli, Valeria Gambacorta, Franco Trabalzini, Laura Dipietro, Giampietro Ricci

**Affiliations:** a University of Perugia, Otolaryngology Department, Perugia, Italy; b Santobono-Posillipon Hospital, Otology and Cochlear Implant Unit, Naples, Italy; c University La Sapienza of Rome, Organ of Sense Department, Rome, Italy; d Meyer University Hospital, Otolaryngology Department, Florence, Italy; e Highland Instruments, Cambridge, United States

**Keywords:** Rachnoid cyst, Surgery, Endoscope, Minimally invasive, Retrosigmoid approach

## Abstract

**Introduction:**

Arachnoid cyst in the internal auditory canal is a quite rare pathology but due to its compressive action on the nerves in this district should be surgically removed. Several surgical techniques have been proposed but no surgeons have used the minimally assisted endoscope retrosigmoid approach for its removal.

**Objective:**

To investigate the feasibility of using a minimally invasive endoscope assisted retro-sigmoid approach for surgical removal of arachnoid cysts in the internal auditory canal.

**Methods:**

Minimally invasive endoscope assisted retrosigmoid approach allows to access to the internal auditory canal through a minimally invasive retrosigmoid approach that combines the use of a microscope and an endoscope. It is performed in six steps: soft tissue step, bone step, dura step, cerebellopontine angle step (performed using an endoscope and a microscope), microscope-endoscope assisted arachnoid cysts removal and closure. We tested minimally invasive endoscope assisted retrosigmoid approach for removal of arachnoid cysts in the internal auditory canal on two human cadaveric heads (specimens) of subjects affected from audio-vestibular disorders and with arachnoid cysts in the internal auditory canal confirmed by magnetic resonance imaging.

**Results:**

The mass was completely and successfully removed from the two specimens with no damage to the nerves and/or vessels in the surgical area.

**Conclusion:**

The results of our study are encouraging and support the feasibility of using minimally invasive endoscope assisted retrosigmoid approach for removal of arachnoid cysts in the internal auditory canal. While further clinical in-vivo studies are needed to confirm the accuracy and safety of using the minimally invasive endoscope assisted retrosigmoid approach for this specific surgery, our group has successfully used the minimally invasive endoscope assisted retrosigmoid approach in the treatment of microvascular compressive syndrome, schwannoma removal and vestibular nerve resection.

## Introduction

Arachnoid cysts (ACs) located in the head are rare; even more rarely (10%) they are located in the internal auditory canal (IAC), in which case they can cause audio-vestibular and/or facial symptoms, depending on the specific nerve that is compressed by the cyst.^1^, ^2^, ^3^, ^4^, ^5^

The symptomatology of ACs in the IAC was first described by Sumner et al. and Thijssen et al.^1^, ^2^ Several years later, Schuktnecht and Gao described three cases of ACs in the IAC. In two cases the cyst compressed the vestibular nerve and the patient suffered from recurrent episodes of vertigo; in the other case the cyst compressed the cochlear nerve and the patient suffered from tinnitus.^3^ In 2018, Ungar et al. analyzed the correlation between ACs in the IAC and symptoms in 22 patients. They found that in most patients the AC was located in the fundus of the IAC and only 13 patients presented with a symptom that might have been caused by the AC. The most common symptom (exhibited by 94% of patients) was a moderate to severe form of sensorineural hearing loss (SNHL) associated with speech discrimination deficit and tinnitus. None of the patients reported vestibular symptoms even though the AC compressed both the cochlear and vestibular nerve.^4^ A meta-analysis on 46 articles conducted by Di Stadio in 2016^5^ showed that ACs caused nerve compression syndromes more commonly than nerve displacement; the cochlear and vestibular nerves were the structures most commonly involved in compressive syndromes while the facial nerve was typically only displaced. Furthermore, growth of AC, whether rapid or slow, might damage the cochlear nerve causing a severe to profound form of SNHL.^2^, ^5^

Currently, surgical removal is the only method to avoid worsening of symptoms caused by ACs in the IAC. Several surgical approaches have been proposed, such as the translabyrinthine,^6^ the posterior fossa,^7^ the middle temporal fossa,^8^ the retro-sigmoid^9^ and the endoscopic approach,^6^, ^10^ all of which have limitations. Surgery risks associated with the posterior, middle temporal fossa, and retrosigmoid approaches, which are the most commonly used approaches, include persistent headaches, brain damage, liquor fistula, and leak of Cerebrospinal Fluid (CSF). The translabyrinthine approach can only be implemented in patients who have already completely lost hearing function as it destroys the cochlea. A fully endoscopic approach limits the number and quality of surgical maneuvers as the surgeon needs to use one hand to hold the endoscope.

Combined approaches such as the endoscope assisted retro-sigmoid approach have been shown to partially mitigate surgery risks. In 2011 Feck et al.^6^ showed that removal of AC in the IAC via this approach was safe and achieved complete resolution of symptoms even though the capsule of the AC could not be completely removed.

In this article we describe the endoscope assisted minimally invasive retro-sigmoid approach (EAMIRSA) for surgical removal of ACs in the IAC. The EAMIRSA has been successfully used by our group and others to treat different IAC pathologies^11^, ^12^, ^13^, ^14^, ^15^ and vascular loop compressions on the facial^14^ and cochlear nerve.^13^ We propose to use the EAMIRSA for AC removal and in this article we describe the steps of the surgical procedure, which we tested on human cadaveric models (specimen) of subjects with AC in the IAC.

## Methods

### Selection of temporal bone specimens

Thirty human head specimens with a positive history of SNHL were analyzed by 1.5 T MRI of the IAC. MRI was acquired with slide thickness 0.7 mm, TE 190 mill sec, TR1700 mill sec, Field of view 100% and Matrix 0/412/337/0. Two specimens that in T2 presented a mass in the IAC compatible with an AC were selected for performing EAMIRSA.

### EAMIRSA for resection of AC from the IAC

**Figure 1:**
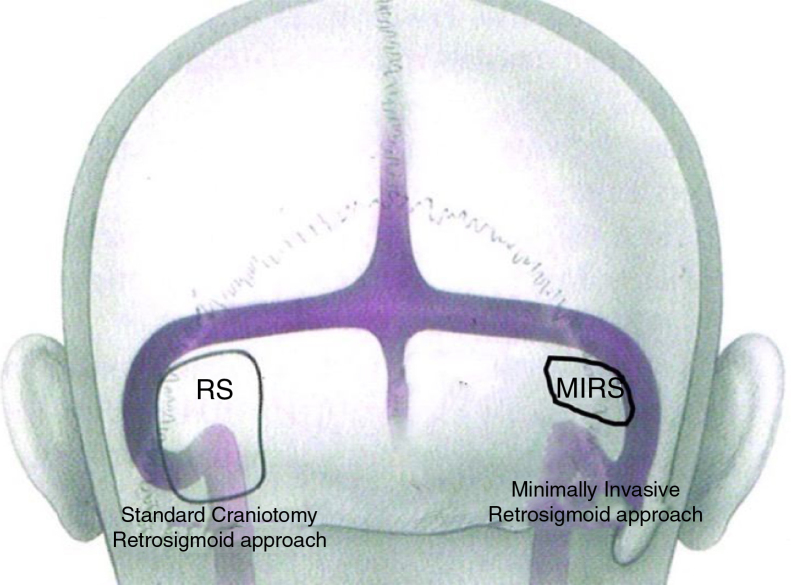
Difference in dimension of a craniotomy performed with a traditional retro-sigmoid approach and with the EAMIRSA.

**Figure 2:**
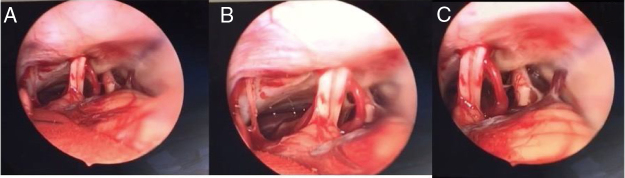
The figures show the nerves and vascular structure in the CPA up to their entry in the bone part of the IAC (the view in A is farther than the one is B; the view in C shows different point views distances from nerve bundle).

The EAMIRSA (Fig. 1) allows access to the IAC through a minimally invasive retro-sigmoid approach that combines the use of a microscope and an endoscope. The endoscope offers a 360° view of the Cerebellopontine Angle (CPA) (Fig. 2) and under microscope view the surgeon can carefully dissect the cyst from the surrounding nerves using both hands. By drilling the wall of the IAC with a retrograde dissection it is possible to completely access the IAC. The EAMIRSA is performed following 6 steps: soft tissue step, bone step, dura step, CPA step (performed using an endoscope and a microscope), microscope-endoscope assisted AC removal and closure.

Image from Ricci GP, Di Stadio A et al. “Endoscope-assisted retrosigmoid approach in hemifacial spasm: an orl experience”. Brazilian Journal Otolarhynolaryngology 2018; pii: S1808-8694(18:30179-4) with agreement and approbation of BJORL.

#### Soft tissue step

**Figure 3:**
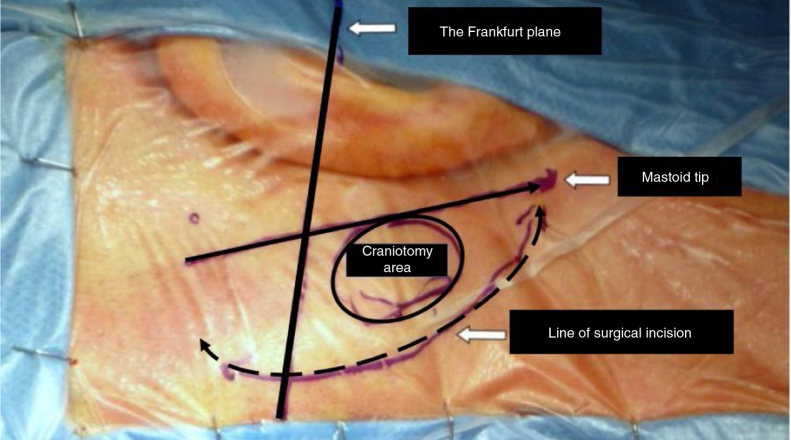
The image shows the area where the minimally invasive craniotomy is performed with the landmark. Image from Ricci GP, Di Stadio A et al. ''Endoscope-assisted retrosigmoid approach in hemifacial spasm: an orl experience''.

After drawing landmark lines as shown in Fig. 3, 10 mL of 2% lidocaine with 1:100,000 epinephrine solutions is injected in the retro-auricular area. A 6–8 cm arciform shaped skin incision extending from the tip of the mastoid to ˜2 fingers behind the helix projection on the retro-mastoid region is performed with a cold scalpel (nº 11).

With an electric scalpel a skin flap is created via careful and soft dissection, moving from the posterior to the anterior part of head; the flap is then elevated from an anterior and superior to a posterior and inferior direction up to the digastric groove. Note this flap needs to be prepared with extreme care to avoid necrosis and thus CSF leak; additionally, it needs to have appropriate thickness to be able to protect the occipital artery and C2 nerve during the closure step (see below) and to allow closure of the craniotomy at the end of the surgery. The flap is then elevated in order to expose the retro-mastoid area to allow visualization of the emissary vein, which will need to be ligated to avoid bleeding. Finally, a Beckman orthostatic retractor is placed so as to allow visualization of the operating field which will be delimited by the posterior edge of the mastoid, the occipital lines superior and inferior and the digastric groove.

#### Bone step

Using the mastoid emissary vein as a landmark, a circular craniotomy of 1.5–2 cm diameter is performed posteriorly to the sigmoid with a large cutting burr first and then with a diamond burr when the sound of the large cutting burr becomes metallic (to preserve the surface of the dura and sigmoid sinus). The dissection needs to be continued until the blue line of the posterior margin of the sigmoid sinus (anterior limit of the dissection) becomes visible. Note the bone dust needs to be preserved during the drilling procedure as it will be needed for obliterating the craniotomy during closure.

#### Dura step

First, anesthesia dosage is increased as deep sedation, reduction of blood pressure, and patient hyperventilation are required (arterial pCO_2_ around 25 mmHg) to allow the CSF intra-cranial pressure to decrease and to induce spontaneous retraction of the cerebellum.

After exposure, a V shape incision is performed with a micro-scalpel in the dura behind the sigmoid, without touching the cerebellum. This incision is performed ˜2 mm from the craniotomy edges in order to facilitate dura re-suturing at the end of surgery.

The duraflap is fastened to the anterior soft tissues with a silk suture; finally, a neurosurgical micro-cotton or a surgical substitute of dura (1.5 × 5 cm) is placed in the area to protect the cerebellum.

#### CPA step (combining microscope and endoscope)

First, dissection is performed under microscopic view. Very carefully the cerebellum is compressed posteriorly in the direction of the inferior part of the craniotomy. Then, the cisterna magna is opened to allow liquor to exit and cerebellum to collapse, which allows access to the CPA and to the internal portion of the IAC. The arachnoid cyst in contact with the cranial nerves (acoustic-facial bundle and lower cranial nerves) is dissected with a smooth micro-dissector and micro-scissors.

At this point, dissection continues using a rigid, 30° angled, 4 mm diameter endoscope, which is carefully inserted, directed downwards to visualize the CPA and the IAC, and specifically the acoustic-facial bundle which is the central landmark in the CPA of the group of nerves running from the cerebellum toward the IAC (Fig. 2). To achieve visualization of these areas the endoscope is maneuvered as follows: first, the tip is placed above the acoustic-facial nerve bundle toward the trigeminal area; then it is slowly moved between the auditory and the glossopharyngeal nerve to visualize the choroid plexus, the root exit zone of the facial, the pica and vertebral artery. The endoscope lens can be cleaned by touching its tip with the cotton patch to avoid having to withdraw and introduce the endoscope into the surgical field multiple times.

Finally, part of the IAC is drilled using a diamond burr in the direction of the bone with slow and small movements to avoid damaging the PCA.

#### AC removal

The IAC is visualized and AC location is identified using the endoscope, which is fixed on a flexible-rigid arm so to allow the surgeon to use the microscope and both hands. Using a micro-dissector, the capsule of the AC is smoothly separated from the compressed nerve(s). The dissection is performed in the direction of the CPA (retrograde) to avoid traction and thus nerve damage and can be conservative if the AC cleavage plan is well preserved. Typically, this is an easy step if the area does not have a history of inflammation.

Following AC removal, one or more Teflon pads (2–3 mm size) can be placed between the nerves to prevent synechia during the healing process. Finally, the endoscope is moved around to inspect the cyst's surrounding structures and check for bleeding.

#### Closure

The neurosurgical micro-cottonoids previously placed on the surgical area are now removed and the CPA is washed with abundant saline solution. The dura is closed with 6/0 single reabsorbable stitches and pieces of subcutaneous fat tissues are inserted between the stitches and fixed with fibrin glue to achieve waterproof closure. A piece of absorbable dural substitute is placed on the dura external surface, and a cover of absorbable hemostat of adequate size is placed to cover the dura.

The bone dust (from the bone step) is mixed with fibrin glue and used to fill the craniotomy until a homogenous coverage of the area is achieved. Finally, extra fibrin glue is added to totally cover the area.

The final closure steps are: 1) Closure of the muscle-periosteal flap with 2/0 interrupted absorbable sutures and 3/0 subcutaneous absorbable stitches; 2) Skin suturing with 2/0 nonabsorbable stitches; and 3) application of a slight compression bandage, to be kept for 4 days.

## Results

Following the procedure above, AC was removed from the two selected specimens using the EAMIRSA. Both procedures were performed by the same surgeon (ADS).

In one case the AC (0.9 mm) was located in the lateral part of the IAC and compressed the cochlear and the superior vestibular nerves; in the other case the AC (1.2 mm) was located centrally in the IAC and dislocated all 4 nerves. The surgery took 1 h and 55 min in the first specimen and 2 h and 15 min in the second specimen. In both cases the AC was removed preserving its external capsule and with complete preservation of all nerves of the IAC.

## Discussion

In this article we described the EAMIRSA procedure for removal of AC in the IAC, step-by-step, and showed that using the EAMIRSA we were able to completely remove the cyst in two specimens. In both cases the surgery was completed in a reasonable time.

AC in the IAC has a symptomatology similar to that of other masses that can be found in the IAC, for example schwannoma, meningioma and lipoma^10^, ^11^ (which are its differential diagnoses); in fact its diagnosis is typically made during surgical procedures.^2^, ^16^, ^17^ These similarities suggest that the EAMIRSA could also be used to remove these other types of tumor.

**Figure 4:**
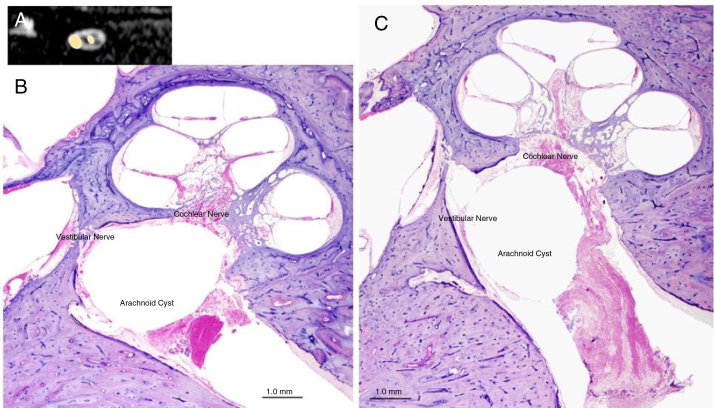
Panel A shows an AC in the IAC (MRI, sagittal view); Panel B and C show the typical aspect of an AC in the IAC of a human temporal bone (microscopic view, otophatology aspect).

**Figure 5:**
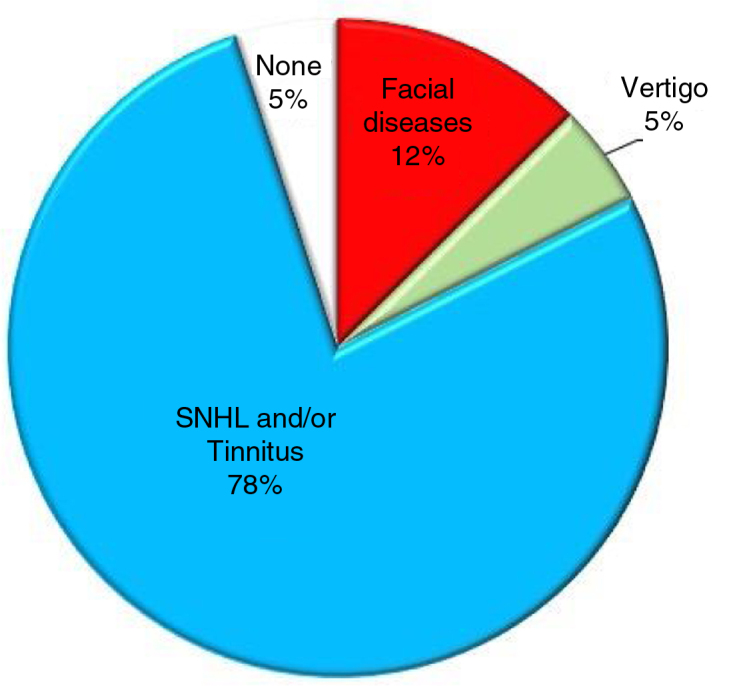
Prevalence of symptoms that may be displayed by patients with AC in the IAC.^1^, ^2^, ^3^, ^4^, ^5^, ^16^, ^17^, ^18^, ^19^, ^20^, ^21^

An AC may be totally asymptomatic if it is small and located centrally in the IAC or may cause specific symptoms if it compresses one or more nerves and is located laterally between the nerves and the internal wall of the IAC^5^ (Fig. 4). The most common symptom is SNHL^3^, ^4^, ^17^, ^18^, ^19^, ^20^, ^21^ which may present with or without tinnitus;^16^, ^19^, ^21^ other symptoms include dizziness, vertigo,^5^, ^17^ Facial Palsy (FP)^1^, ^2^ and hemifacial spasm^21^, ^22^ (Fig. 5). As multiple nerves (acoustic, vestibular, facial) can be involved, the ideal surgical approach preserves nerve function, has minimal post-surgical complications, and allows complete excision of the mass. In the EAMIRSA this is achieved by combining the use of an endoscope and a microscope, an approach that builds on the minimally invasive retro-sigmoid approach first proposed by Bremond et al. in 1974 ^23^ and further developed in 1993.^23^, ^24^, ^25^

Image O from “Di Stadio A. Arachnoid cyst of internal auditory canal: how the temporal bone findings explain ear symptoms and suggest the best treatment to do. Otolaryngol Open J. 2016;2(5): 125-131″ with agreement and approbation of the Otolaryngology Open. J.

In surgeries of the CPA and IAC, the EAMIRSA allows maintenance of normal intracranial pressure, limits the chance of bacterial infection, CSF leaks, and persistent headaches commonly observed when the traditional retro-sigmoid approach is used.^14^ The use of the endoscope is key to achieve these superior outcomes as it allows a 360° view of structures’ relative positions^14^, ^23^, ^24^, ^25^ without mechanical retraction of the cerebellum. Compared to the traditional microscope approach,^25^, ^26^, ^27^ the EAMIRSA offers a better vision (360° view of the surgical field as opposed to a straight linear view) and a less traumatic approach.^6^, ^8^ Compared to a fully endoscopic approach, the EAMIRSA offers better control of bleeding and easy switching to a traditional surgery under microscope should intra-operative complications arise.^28^, ^29^

In the surgical treatment of ACs in the IAC, the EAMIRSA has also distinct advantages. A minimally invasive retro-sigmoid approach that uses only a microscope would not be feasible, due to the anatomy of the posterior fossa which makes it difficult to visualize the entire course of the nerves running in the IAC and in the *porus* portion.^26^, ^27^, ^28^ Conversely, by accessing directly the surgical site the endoscope permits good visualization of the relative position of the AC, nerve and IAC; this improved visualization allows more precise surgical maneuvers, reduces the risk of neurologic complications^13^, ^14^ and eliminates the need for an extended craniotomy. During the AC removal phase of the procedure, combined use of microscope and endoscope allows the surgeon to have a full view of the surgical area (through the endoscope) and to perform bimanual surgical maneuvers (through the microscope).

The EAMIRSA has been successfully used by our group for treating hemifacial spasm^14^ and tinnitus^13^ caused by loops in the IAC compressing the nerve(s), removal of vestibular schwannoma^11^ and vestibular neurectomy^11^ without relevant immediate^13^ and long term post-operative sequelae.^14^ The EAMIRSA was used even in elderly subjects (who have a higher risk of complications due to associated comorbidities such as hypertension) without increased postoperative sequelae.^14^

Limitations of the EAMIRSA when used in the CPA and IAC include a relative slow learning curve and risk of damaging nerve and/or vascular structures due to the use of an endoscope in such small areas. Additional in vivo studies are needed to confirm efficacy and safety of this approach.

## Conclusion

We have initiated use of the EAMIRSA has for surgical treatment of pathologies of the CPA and IAC. This article described how to use this approach, step-by-step, for the treatment of symptomatic ACs in the IAC. Performance of surgery using the EAMIRSA requires understanding of anatomy, ability to drill CPA and IAC structures acquired through temporal bone dissection training, and microsurgery skills.

## Conflicts of interest

The authors declare no conflicts of interest.
